# Aspects of Fe-Incorporation into CaTiO_3_-SrTiO_3_ Perovskites and Their Catalytic Application for Ammonia SCO/SCR

**DOI:** 10.3390/molecules29235603

**Published:** 2024-11-27

**Authors:** Paulina Gwóźdź, Agnieszka Łącz, Sylwia Górecka, Kateřina Pacultová, Kamil Górecki, Lucie Obalová, Ewa Drożdż

**Affiliations:** 1 Department of Inorganic Chemistry, Faculty of Materials Science and Ceramics, AGH University of Krakow, al. A. Mickiewicza 30, 30-059 Krakow, Poland; 2 Institute of Environmental Technology, Centre for Energy and Environmental Technologies, VSB-Technical University of Ostrava, 17. Listopadu 2172/15, 708 00 Ostrava-Porub, Czech Republic

**Keywords:** perovskites, iron, ammonia oxidation, NOx reduction, XANES

## Abstract

Perovskite materials in the CaTiO_3_-SrTiO_3_ system doped with different amounts of iron (1, 2 and 5 mol.%) and various Ca/Sr ratios were prepared by the modified citrate method. Additionally, the materials with 0.05 deficiency in strontium/calcium sublattice and 5 mol.% of Fe were also synthesised. The materials were subjected to structural (XRD, XANES) and microstructural (SEM) characterisation, as well as the analysis of susceptibility to reduction/oxidation processes. The structural analysis indicates a lack of iron-containing phases; thus, an incorporation of Fe into the perovskite structure was postulated. Additionally, the oxidation state of iron in the perovskite structure changes with the dopant amount. The temperature-programmed reduction measurements showed partial reversibility of the reduction processes. For the materials with the highest iron amount, the catalytic tests in NH_3_-SCO and NH_3_-SCR reactions were carried out. The materials showed high catalytic activity and high selectivity to N_2_ in the NH_3_-SCR process; however, they were inactive in NH_3_-SCO.

## 1. Introduction

Environmental catalysis is essential to addressing global challenges related to pollution and sustainable development. Catalysts are designed to facilitate chemical reactions that reduce harmful pollutants, thereby reducing the emission of toxic gases such as nitrogen oxide (NO) and ammonia (NH_3_) from industrial processes and vehicles. Advances in this field improve energy conversion efficiency and promote cleaner production techniques, mitigating environmental impact. Innovative materials and catalytic processes enhance the efficient use of natural resources, reducing dependence on non-renewable sources. Key processes such as selective catalytic reduction of nitrogen oxides with ammonia (NH_3_-SCR) and selective catalytic oxidation of ammonia (NH_3_-SCO) are crucial for reducing air pollution. In the NH_3_-SCR process, the reduction of nitrogen oxides (NO_x_) to nitrogen (N_2_) and water (H_2_O) in the presence of ammonia (NH_3_) and a catalyst occurs, effectively controlling NO_x_ emissions, while in the NH_3_-SCO process an oxidation of residual ammonia to nitrogen (N_2_) and water (H_2_O), preventing secondary pollution, takes place. The combination of NH_3_-SCR and NH_3_-SCO processes provides comprehensive emission control, with NH_3_-SCR reducing NO_x_ emissions and NH_3_-SCO ensuring the conversion of unreacted ammonia from the SCR process into harmless substances. Perovskite materials with a general ABO_3_ formula have proven to be promising candidates in environmental catalysis due to their unique structural properties and high catalytic activity [1,2,3,4]. These materials are effective in various applications, including SCR and SCO processes. One method of modifying the properties of perovskites is doping, which involves introducing ions with different valence states into the perovskite lattice in place of the host ion. The doping process can alter the crystal structure, electron band configuration, and defect chemistry, enhancing or modifying the material’s functional properties [5,6,7,8,9]. To ensure minimal lattice distortion and maintain structural integrity, dopant ions should have ionic radii similar to ionic radii of the host ions [10] Doping with heterovalent ions, including d-block elements such as manganese (Mn), iron (Fe), cobalt (Co), nickel (Ni) and chromium (Cr), can lead to significant changes in conductivity, concentration of electric charge carriers, and stability of perovskite compounds [11,12,13,14,15,16,17,18,19,20,21,22,23,24] The use of d-block element dopants is advantageous due to their variable oxidation states and ability to participate in mixed ionic–electronic conductivity, greatly enhancing catalytic properties [15,25]. The unique structural and electronic characteristics introduced by these dopants facilitate efficient electron transfer through rapid oxidation state changes and redox reactions, which accelerates surface reactions and improves catalytic effectiveness. Moreover, the oxygen vacancies created by the dopants aid in lattice oxygen transport and allow for direct interaction with gaseous reactants like NO_x_ and CO_2_ [26]. These factors suggest that the catalytic reactions on doped perovskite surfaces follow the Mars-van Krevelen (MvK) mechanism, which depends on the interplay between lattice oxygen, oxygen vacancies and gaseous reactants for effective catalysis.

The use of doped perovskites as catalysts has gained increasing attention in recent years. While most studies have focused on their photocatalytic applications, there is a growing need to explore their potential in other catalytic processes, particularly in reactions like NH₃-SCR and NH₃-SCO, where catalytic materials must meet stringent criteria of efficiency, safety and cost-effectiveness. The search for materials that are safe for living organisms and do not rely on expensive noble metals has driven researchers to investigate alternative dopants and catalytic systems. Our promising results from previous studies on Co- and Cu-doped SrTiO₃ in NH₃-SCR and NH₃-SCO reactions [4] motivated us to explore the potential of Fe-doped perovskite-based materials. The choice of iron was guided by several key considerations:

(i) Redox Versatility and Catalytic Properties: Iron, like cobalt, can adopt multiple oxidation states (Fe^2^⁺/Fe^3^⁺/Fe^4+^) that facilitate redox reactions crucial for catalytic activity. Similar findings have been reported in the literature, where Fe-doped perovskites exhibited enhanced catalytic performance due to efficient electron transfer and redox cycling [27].
(ii) Higher Incorporation Capacity: Iron ions are more readily incorporated into the perovskite lattice [28] compared to cobalt or copper, as indicated in previous studies [19]. This higher solubility can lead to a greater density of active sites and better stability under reaction conditions.
(iii) Economic and Environmental Advantages: Iron compounds are significantly more cost-effective than cobalt or copper compounds. Additionally, iron compounds are less harmful to the environment and living organisms, aligning with the global push for sustainable and environmentally friendly catalysts. Examples in the literature highlight iron-based perovskites as promising candidates for industrial applications due to their low toxicity and abundance [29].
(iv) Defect Engineering and Adsorption Sites: The incorporation of iron ions into the ABO₃ perovskite lattice is known to create oxygen vacancies and other point defects, similar to the effect observed with cobalt doping. These defects can serve as adsorption and activation sites for reactants, enhancing catalytic efficiency [27,30,31].

By leveraging these unique features, iron-doped perovskites offer a compelling alternative to Co- and Cu-based systems, providing a balance of performance, cost and environmental safety. This work builds on previous studies and seeks to further understand the role of iron in tuning the structural and catalytic properties of perovskite-based materials.

So far, iron-doped perovskite materials have primarily been studied for applications in electrochemical devices, such as solid oxide fuel cells (SOFCs) [14,32], or in photocatalytic reactions [33,34]. However, our research focuses on investigating the catalytic activity of a mixed CaTiO_3_-SrTiO_3_ perovskite system doped with Fe in the Ti sublattice, as, to our knowledge, this type of binary system has not been used so far as a support (and at the same time as a catalyst) for environmental catalysis applications.

Due to the similarity in ionic radii between the host ion, titanium (Ti), and the dopant, iron (Fe), iron can be incorporated into the B-site (Ti site) of the perovskite structure both in the case of introducing iron into the CaTiO_3_ and SrTiO_3_ structure. Titanium exhibits octahedral coordination (coordination number CN = 6) in the ABO_3_ structure and, in its +IV oxidation state, has an ionic radius of 0.605 Å [35]. When iron is introduced into the B-site, it can occupy the site in either the +II, +III and/or +IV oxidation state. However, Fe^3^⁺ (high-spin, ionic radius 0.645 Å [35]) and Fe^4+^ (ionic radius 0.585 Å [35]) are both size-compatible with Ti^4+^, facilitating their incorporation into the perovskite lattice. In perovskite structures, Fe^2^⁺ and Fe^3^⁺ typically exist in high-spin configurations due to crystal field stabilisation [36]. However, Fe^2^⁺, with a high-spin ionic radius of 0.780 Å [35], is larger and less likely to substitute directly for Ti^4+^ but may still incorporate into the B sublattice under reducing conditions [35]. The amount of dopant that can be introduced into the perovskite structure is limited and depends on various factors. Nonetheless, research has shown that the efficiency of dopant incorporation can be improved by introducing a deficiency in the A sublattice of the perovskite (non-stoichiometry) [37,38,39]. This A-site non-stoichiometry creates vacancies that promote the incorporation of higher concentrations of dopants, enhancing the material’s catalytic and electrical properties [40,41]. Therefore, for our studies, we obtained the stoichiometric materials (in which the number of moles of ions in the A sublattice is equal to the number of moles of atoms in the B sublattice), and the materials with non-stoichiometry introduced at the synthesis stage (with a reduced number of A ions in relation to B ions). Thus, in the presented manuscript, for the first time, the research on the analysis of the possibilities of using materials in the SrTiO_3_-CaTiO_3_ system doped with iron (with preserved stoichiometry and non-stoichiometric ones) as catalysts for selected environmental reactions is presented.

## 2. Results and Discussion

The nominal composition of the Fe-modified materials based on the CaTiO_3_-SrTiO_3_ system, together with the proper labels, is presented in Table 1. In the notation used for these materials, the symbol s indicates a stoichiometric A-site composition, meaning there is no intentional deficiency in the total (Sr + Ca) content. Conversely, the symbol n represents a non-stoichiometric A-site composition, where a 5% deficiency in the (Sr + Ca) content was introduced (A-site occupancy of 0.95). The numerical values following these symbols correspond to the molar ratio of Fe dopant relative to titanium.

**Figure 1:**
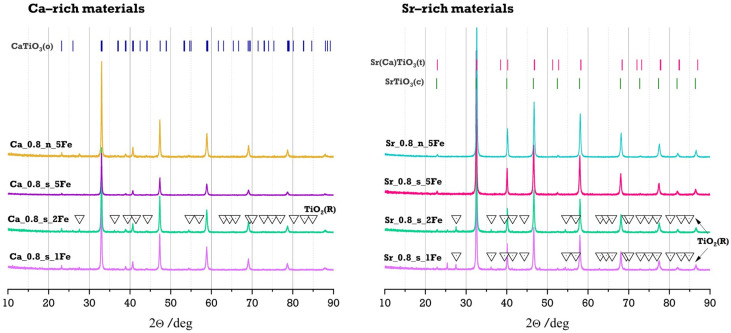
The diffraction patterns for Fe-modified Ca-rich and Sr-rich with the patterns for cubic SrTiO_3_ (Pm-3m, ICCD No. 98-005-6717), tetragonal Sr_0.65_Ca_0.35_TiO_3_ (I4/mcm, ICCD No. 98-009-4572) and orthorhombic CaTiO_3_ (Pnma, ICCD No. 98-016-2910).

The phase composition of Fe-modified and reference materials was determined through X-ray diffraction (XRD) and calculated using the Rietveld method. Diffractograms for Ca-rich and Sr-rich materials are presented in Figure 1, and the phase compositions are summarised and presented in Table 2.

All synthesised materials exhibited a perovskite-type structure as the main or the only phase. The small amounts of additional phase as TiO_2_ (rutile, P42/Mnm, ICCD No. 98-008-5492) for Fe-modified Ca-rich and Sr-rich samples containing 1% and 2% of iron were detected. The amount of additional TiO_2_ phase is higher for Sr-rich samples (approx. 4%) than for Ca-rich samples (up to 1.4%). All samples with 5% Fe content (both stoichiometric and those with assumed non-stoichiometry in sublattice A) show only the presence of the perovskite phase; however, depending on the Ca/Sr ratio, these are phases with different symmetry. For Ca-rich reference materials, an orthorhombic phase (CaTiO_3_, Pnma, ICCD No. 98-016-2910) was identified, whereas Sr-rich materials exhibited phases with higher symmetry: tetragonal (Sr_0.65_Ca_0.35_TiO_3_, I4/mcm, ICCD No. 98-009-4572) and cubic (SrTiO_3_, Pm-3m, ICCD No. 98-005-6717). Generally, the introduction of iron into the Ca-rich system did not affect the phase symmetry—all samples retained the orthorhombic symmetry, characteristic for the undoped CaTiO_3_ structure (CaTiO_3_, Pnma, ICCD No. 98-016-2910). However, some changes in the symmetry of the iron-doped systems can be observed for the Sr-rich series compared to the symmetry of the undoped materials. Introducing a small amount of iron into the Sr_0.8_Ca_0.2_TiO_3_-based system leads to an increase in the amount of tetragonal phase compared to the phase composition of Sr_0.8_Ca_0.2_TiO_3_ (presented in [42]). This reduction in symmetry can be attributed to the distortion of the TiO_6_ octahedra caused by the incorporation of Fe ions, which have different ionic radii and valence states compared to the Ti^4+^ ions which they replace. Additionally, the Jahn–Teller effect, associated with Fe^2+^ in its high-spin configuration, contributes to this distortion by degenerating specific orbitals (particularly the dz^2^ and dx^2^-y^2^ orbitals), further reducing the symmetry of the perovskite structure. However, with the increase in the amount of Fe in the system (from 1 mol.% up to 5 mol.%), the amount of cubic phase increases. It can be a result of changing the oxidation state of iron towards a higher (+4) oxidation state, which stabilises the perovskite structure since Fe^4+^ is isovalent with host Ti^4+^ ions. A similar behaviour, changing the oxidation state of the dopant with its amount, was previously observed for Co-modified SrTiO_3_ [19]. In the case of the Ca-rich system, we did not observe the same reduction in symmetry. This can be attributed to the fact that the Ca-rich perovskite system already adopts a lower-symmetry orthorhombic structure, which is more distorted compared to the tetragonal or cubic phases. As a result, further doping with iron in the Ca-rich system does not induce a significant additional distortion to the lattice that would drive a transition to a lower-symmetry phase. The orthorhombic phase is already well adapted to accommodate the dopants, and thus the symmetry remains largely unaffected. This behaviour highlights the difference in how dopants interact with higher-symmetry versus lower-symmetry perovskite phases, where the higher-symmetry phases (observed for Sr_0.8_Ca_0.2_TiO_3_-based system) are more likely to undergo symmetry reduction due to dopant incorporation.

For all obtained materials, both Ca-rich and Sr-rich series, no Fe oxide phases (FeO, Fe_2_O_3_, or Fe_3_O_4_) were detected in the XRD data. This suggests that these phases are either below the detection limit or present in an amorphous form. Nonetheless, the XRD analysis confirmed the successful incorporation of Fe into the perovskite structure for both Ca-rich and Sr-rich systems.

**Figure 2:**
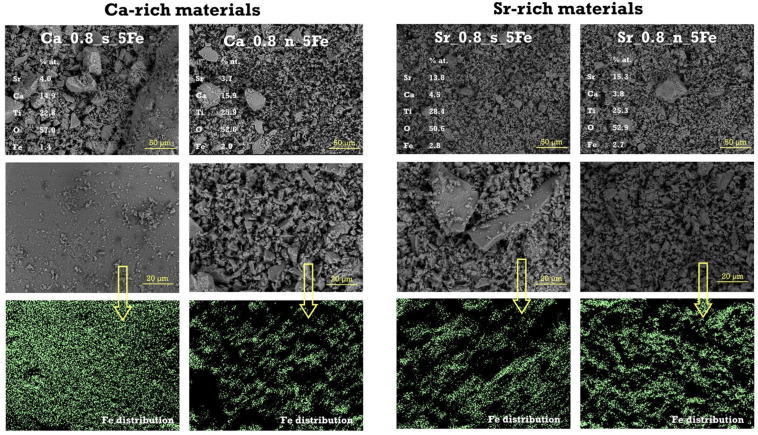
SEM microphotographs (recorded using SE + BSE detector), along with the average chemical composition (EDS results) and the distribution of Fe (EDS mapping mode) for Ca-rich and Sr-rich materials modified with 5 mol.% of Fe.

The SEM images of Ca-rich and Sr-rich materials modified with 5% of Fe (Figure 2) indicate that all samples exhibit similar microstructures. EDS results, based on an average of five measurements taken from different areas of each sample, generally align with the assumed material compositions. However, given the relatively limited accuracy of the EDS method, these values should be interpreted with caution in quantitative terms. Despite this, the EDS mapping clearly demonstrates that iron is uniformly and homogeneously distributed within the Fe-modified Ca-rich and Sr-rich materials, with no noticeable agglomerations or inclusions. This suggests the absence of Fe-rich phases (e.g., iron oxides), which is consistent with the results of the XRD analysis.

**Figure 3:**
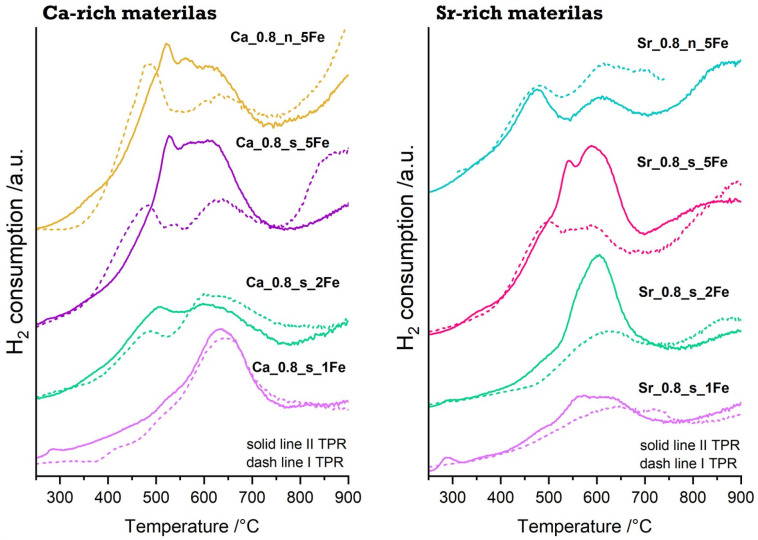
I TPR and II TPR profiles for Fe-modified Ca-rich and Sr-rich materials.

The temperature-programmed reduction measurements for Ca-rich and Sr-rich materials were carried out for samples after calcination (the first reduction process—I TPR) and for samples subjected to an oxidation process (after I TPR). The second TPR was conducted to evaluate the reversibility of reduction–oxidation processes in the systems. The I and II TPR profiles shown below (Figure 3) are not identical for individual materials, but they have reduction maxima in the same temperature range of 350–700 °C. Differences in the shape of reduction profiles may result not only from the nature of the material but also from the oxidation conditions used, as calcination is a process carried out in an air atmosphere, whereas controlled oxidation (TPOx) is a process using a mixture containing only 5% O_2_ in argon. The reduction effects correspond to the reduction of iron incorporated into perovskite structure as neither the results of X-ray measurements nor the EDS elemental maps indicate the presence of additional, iron-containing phases (e.g., Fe_2_O_3_). The complexity of the reduction peaks indicates the possibility of adopting different oxidation states by iron after being incorporated into the CaTiO_3_-SrTiO_3_ system. Taking into account the ionic radius of iron ions in octahedral coordination: 0.585 Å, 0.645 Å, 0.55 Å, 0.78 Å and 0.61 Å for Fe^4+^, Fe^3+^ (high-spin), Fe^3+^ (low-spin), Fe^2+^ (high spin) and Fe^2+^ (low-spin), respectively, iron, when incorporated into the perovskite structure, can take the place of titanium (the ionic radius of Ti^4+^ is equal to 0.605 Å), mainly adopting higher oxidation states. This is consistent with the TPR profiles since several reduction maxima most likely indicate the occurrence of several reduction processes. Moreover, attention should be paid to the partially repeatable nature of the reduction processes as II TPR profiles indicate at least a partial possibility that the iron present in the perovskite structure will revert to higher oxidation states during TPOx and then be reduced again to iron in lower oxidation states during the second reduction process. However, at least a partial reduction of iron incorporated in the perovskite structure to the metallic form cannot be excluded.

**Figure 4:**
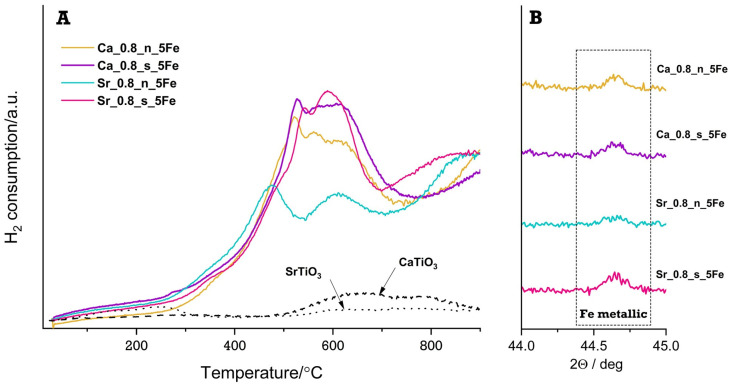
II TPR profiles for Fe-modified Ca-rich and Sr-rich materials and reference samples (SrTiO_3_, CaTiO_3_) (**A**). The diffraction patterns of the materials after the II TPR—the most intense peak in the metallic Fe pattern (**B**).

To confirm the hypothesis of iron reduction to a “0” oxidation state, a diffraction analysis was performed for Ca-rich and Sr-rich materials with the highest iron content (5 mol.%) after the second reduction cycle (II TPR) (Figure 4B). The results of this analysis indicate the presence of small amounts of iron in all tested samples but that the analysis of diffraction data using the Rietveld method did not allow, at an acceptable level of the “goodness of fitness” coefficient, for an unambiguous determination of the amount of metallic iron. To extend the discussion on the course of reduction processes in materials with the highest amount of iron, the II TPR results for these samples were compared with the II TPR results for the reference samples (SrTiO_3_ and CaTiO_3_ are presented in Figure 4A). One can see that the reduction process on the reference profiles is insignificant compared to the reductive effects of iron-doped samples. Moreover, it can be observed that the TPR profiles are similar to each other except for the profile obtained for the Sr_0.8_n_5Fe sample, for which the maxima have lower intensity compared to the maxima for the other materials, and the first maximum occurs at a lower temperature than the first maximum of the other samples. It is worth noting that, for this material, the amount of iron observed on diffraction data is the smallest of all samples. This effect may be due to the larger amount of iron in the +4 oxidation state in the Sr_0.8_n_5Fe material compared to the other materials, which results in a smaller amount of iron reduced to the lowest oxidation state in the same measurement time. However, the oxidation state of the iron cannot be determined based on these results alone. To obtain more detailed insights into the oxidation state of Fe in the materials, X-ray absorption spectroscopy (XAS) was carried out in the XANES (X-ray Absorption Near Edge Structure) region. Combining XAS with the temperature-programmed reduction (TPR) method allows a more comprehensive understanding of the form of iron species present and the specific oxidation states that iron assumes in analysed Fe-modified Ca-rich and Sr-rich materials.

**Figure 5:**
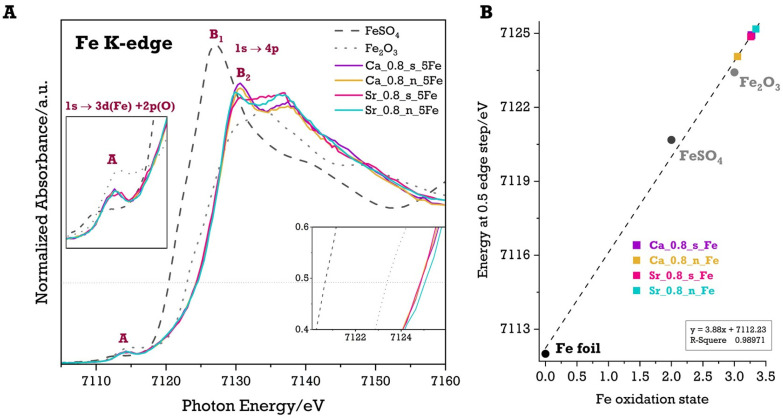
Normalised Fe K-edge XANES spectra of Fe-modified systems and the Fe standards (**A**). Linear correlation plot generated for the determination of the Fe oxidation state, based on the XANES region using a half-edge step method (**B**).

The oxidation states of Fe ions in materials modified with 5 mol.% of Fe were examined using X-ray Absorption Near Edge Structure (XANES) analysis. Additionally, the impact of A-site (Sr/Ca) deficiencies on the efficiency of Fe incorporation into Ti^4+^ sites, as well as the resulting oxidation state of iron, was explored. One of the key advantages of XANES is its ability to correlate the position of the absorption edge with the average oxidation state of d-block elements, where a shift to higher energies indicates a higher oxidation state. Moreover, the overall shape of the XANES spectrum—including the intensity and position of peaks and troughs—provides detailed insights into the local environment of the absorbing element. To estimate the oxidation states of Fe ions in the modified materials, Fe K-edge XANES spectra were recorded and compared to reference standards for Fe^2+^ (FeSO_4_) and Fe^3+^ (Fe_2_O_3_), providing a basis for evaluating the valence state of Fe in both Ca-rich and Sr-rich perovskite systems (Figure 5A).

All compounds exhibited a small absorption peak (A) in the pre-edge region, around 7114 eV. An expanded view of the pre-edge region of the spectra is shown in Figure 5A (left inset). The weak pre-edge peaks correspond to a transition from the deep core 1s level to a quasi-bound state with atomic 3d character. According to the electric dipole moment approximation, this transition is typically forbidden in an ideal octahedral geometry. However, the transition becomes partially allowed when mixing between the 3d and 4p orbitals occurs in non-centrosymmetric or distorted environments [43,44,45,46]. Therefore, the presence of slightly distorted octahedral FeO_6_ sites (Oh) or relatively non-centrosymmetric sites, such as tetrahedral sites (Td), can be inferred based on the analysis of these small absorption peaks. This trend is observed in the standards (FeSO_4_ and Fe_2_O_3_). For FeSO_4_, where iron is in octahedral coordination [47], a less intense peak is observed, shifted towards lower energies. In contrast, for Fe_2_O_3_, where Fe^3^⁺ ions are distributed in both octahedral (Oh) and tetrahedral positions (Td) [44], the intensity of the pre-edge peak (A) is higher and shifted towards higher energies. This shift is related to the presence of Fe^3^⁺ in lower symmetry and tetrahedral coordination, which induces a distortion. However, for the Fe-modified Ca-rich and Sr-rich materials, the changes in absorption in the pre-edge region are too small to allow for a clear analysis of the symmetry changes in the iron coordination environment within these materials. This suggests that any distortion or site occupancy of Fe in these doped systems may be subtle and not easily detected through pre-edge analysis alone. Additional characterisation methods, such as extended X-ray absorption fine structure (EXAFS), may be needed to gain deeper insight into the local structural environment of Fe in these systems.

The main edge peak (features B_1_, B_2_) appears in the absorption energy range between 7130 and 7137 eV, corresponding to dipole transitions from 1s(Fe) to 4p(Fe) orbitals [43,44,45,46]. For the FeSO_4_ standard, we observe a single main edge peak (B_1_), which is shifted towards lower energies. However, for the Fe₂O₃ standard and the Fe-modified Ca-rich and Sr-rich materials, two prominent absorption peaks (B_2_, B_3_) are observed. In the case of Fe_2_O_3_, the presence of two species of Fe ions, which absorb X-rays in tetrahedral (Td) and octahedral (Oh) sites, results in two distinct 1s→4p transitions occurring at different energy ranges [44]. The occurrence of two large absorption peaks for the Fe-modified Ca-rich and Sr-rich materials could suggest the presence of two different forms of iron ions (existing in different coordination symmetries) in these materials. The separation and intensity of the B_2_ and B_3_ peaks could indicate a combination of Fe ions occupying different coordinated sites, similar to what is seen in Fe_2_O_3_. In such a case, the difference in local symmetry around the Fe ions leads to distinct energy levels for the 4p orbitals, reflected in the energy separation between these peaks. The presence of two peaks could also be linked to subtle distortions in the local environment, where Fe ions in slightly different symmetries give rise to distinct 1s→4p transitions.

To emphasise the differences in absorption edge positions between the Fe-modified materials and standards (FeSO_4_, Fe_2_O_3_) and to determine the average oxidation state of Fe ions in the studied materials, an enlargement was presented at a normalised edge height of 0.5 (right inset, Figure 5A). Based on the specific energy position at the midpoint of the edge step for known oxidation states of iron in the standards and the edge energy for Fe foil, a linear correlation was established to estimate the average oxidation state of iron ions in the studied materials. This procedure serves as a tool for evaluating the impact of calcium content and A-site non-stoichiometry on the average oxidation state of Fe ions in the perovskite system. The resulting linear correlation is shown in Figure 5B. The findings suggest that the average oxidation state of Fe ions in all Fe-modified materials is greater than +3, indicating the presence of Fe ions in both +3 and +4 oxidation states (though a minor presence of Fe^2+^ ions cannot be excluded). For the stoichiometric Fe-modified materials, both Ca-rich and Sr-rich, the average oxidation state of iron is very similar, suggesting a comparable distribution of Fe^3+^ and Fe^4+^ ions (and possibly some Fe^2+^) in these materials. The largest differences were observed in materials with A-site non-stoichiometry (Ca_0.8_n_5Fe and Sr_0.8_n_5Fe). The sample with higher calcium content exhibits the lowest average oxidation state of iron ions, indicating the highest proportion of Fe^3+^ (and potentially Fe^2+^) ions to Fe^4+^ ions. Considering the possibility of Fe^2+^ ions in this material, it is worth noting that Fe^2+^ ions in perovskite materials tend to adopt a high-spin configuration, with an ionic radius of 0.78 Å in octahedral coordination, making them larger than Ti^4+^ ions in the same coordination (0.605 Å). Therefore, some Fe^2+^ ions may occupy A-site positions (Ca/Sr) in the perovskite structure. This is even more likely in the Ca-rich system due to the smaller ionic radius of Ca^2+^ ions (1.34 Å [35]) than Sr^2+^ ions (1.44 Å [35]). In contrast, the Sr_0.8_n_5Fe material exhibits the highest average oxidation state of iron, suggesting the largest proportion of Fe^4+^ ions relative to Fe^3+^ (and possibly Fe^2+^). However, this difference is not significant compared to the stoichiometric materials (Ca_0.8_s_5Fe, Sr_0.8_s_5Fe).

**Figure 6:**
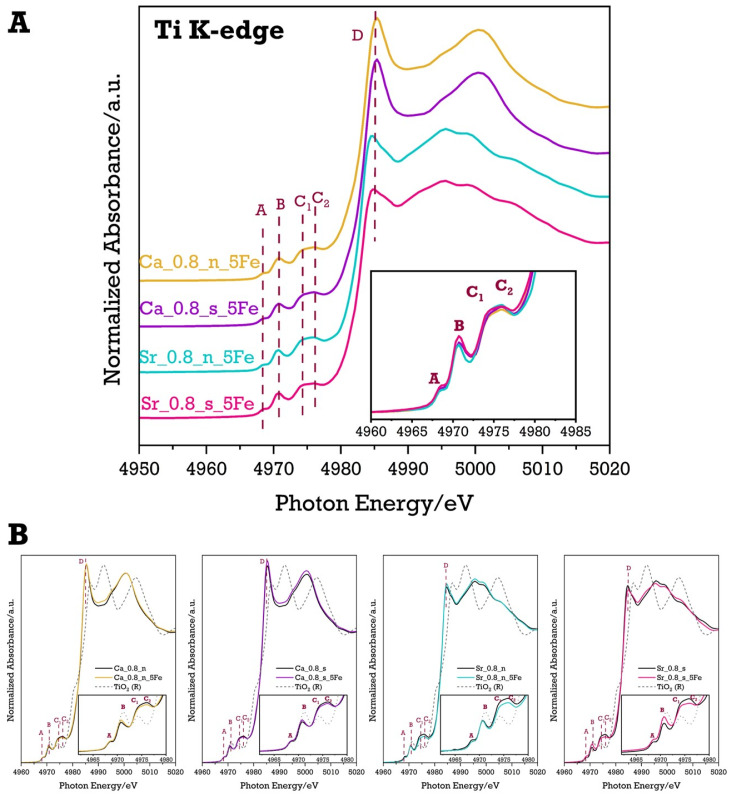
Normalised Ti K-edge XANES spectra of Ca-rich materials and Sr-rich materials (**A**) along with comparisons of reference and Fe-modified systems (**B**).

To confirm the incorporation of iron ions into the B site of the perovskite structure and to complement the information obtained from the Fe K-edge spectra for Fe-modified Ca-rich and Sr-rich samples, the XANES technique was also applied to record Ti K-edge spectra. In perovskites, titanium is in octahedral coordination (TiO_6_ units), and lower structural symmetry causes greater distortion of these units [48]. The Ti K-edge XANES spectra for Ca-rich and Sr-rich materials are shown in Figure 6A.

To better observe the impact of iron modification on the Ti spectrum shape, the recorded spectra for Fe-modified materials (Ca_0.8_s_5Fe, Ca_0.8_n_5Fe, Sr_0.8_s_5Fe, and Sr_0.8_n_5Fe) were compared with reference materials—Sr_0.2_Ca_0.8_TiO_3_ (labelled as Ca_0.8_s), (Sr_0.2_Ca_0.8_)_0.95_TiO_3_ (labelled as Ca_0.8_n), Sr_0.8_Ca_0.2_TiO_3_ (labelled as Sr_0.8_s), and (Sr_0.8_Ca_0.2_)_0.95_TiO_3_ (labelled as Sr_0.8_n) (Figure 6B). The comparison also included the Ti K-edge XANES spectrum for TiO_2_ (rutile). A detailed view of all Ti pre-edge regions for each spectrum is shown in the inset, highlighting pre-edge electronic transitions labelled A, B, C_1_ and C_2_. Assigning the pre-edge peaks is complex due to differing explanations in the literature [49,50,51,52,53,54]. We followed the interpretation that feature A arises from a quadrupole transition (1s to 3d(t_2g_), while peak B is a dipole transition (1s to 4p) with some quadrupole contribution (1s to 3d(e_g_)). Peaks C_1_ and C_2_ represent dipole excitations of 1s electrons into t_2g_ and e_g_ orbitals of neighbouring TiO_6_ octahedra [52,53]. The main edge peak (D) corresponds to a dipole transition from 1s to unoccupied 4p states [54].

For both Ca- and Sr-rich materials, the addition of Fe does not shift the energy of the Ti K-edge absorption, confirming that Ti remains in the +4 oxidation state in all samples. Although introducing Fe into the titanium position does not significantly alter the main edge peak (D), changes in the pre-edge region can be observed due to the presence of Fe (Figure 6A). Specifically, an increase in the intensity of peak B is noticeable in all Fe-modified samples compared to their unmodified reference systems. This increased intensity of feature B suggests greater disorder in the TiO_6_ units in the Fe-modified compounds, likely due to the displacement of Ti^4+^ cations and partial substitution of Ti^4+^ by Fe ions. The rise in feature B is particularly prominent in the Sr-rich stoichiometric material (Sr_0.8_s_5Fe). The analysis of the Ti K-edge spectra confirms the successful incorporation of Fe into the B site of the perovskite structures. This result aligns with findings from temperature-programmed reduction (TPR) and X-ray diffraction studies, which show no evidence of iron in other forms, such as iron oxides.

**Figure 7:**
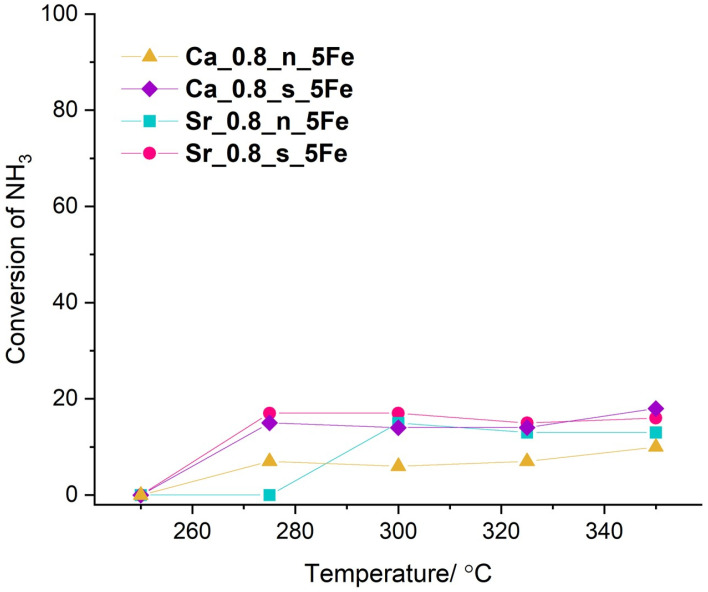
Conversion of ammonia in NH_3_-SCO catalytic tests performed on Fe-modified Ca-rich and Sr-rich materials.

**Figure 8:**
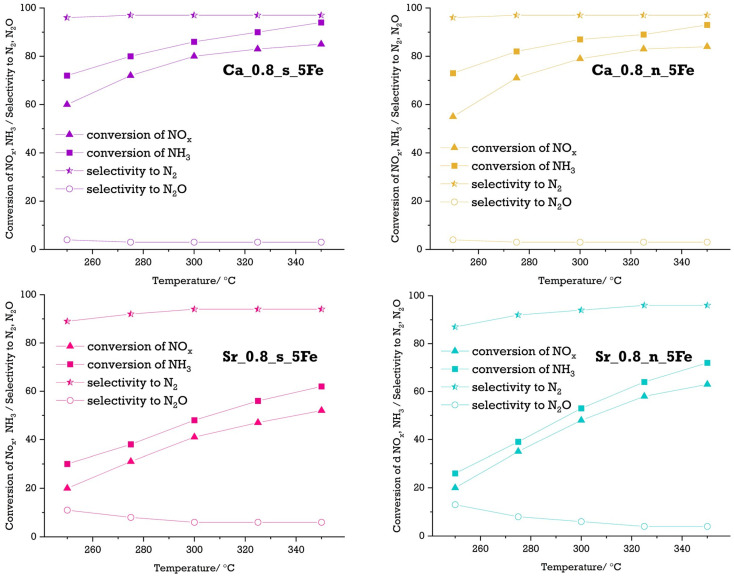
Conversion of NH_3_ and NO_x_ in NH_3_-SCR catalytic tests performed on Fe-modified Ca-rich and Sr-rich materials together with the selectivity to N_2_ and N_2_O.

Materials with 5 mol.% of Fe were selected for catalytic tests since XRD confirmed that the perovskite phase was the only phase present, with no segregation of TiO_2_ detected. The catalytic properties of materials modified with 5 mol.% of Fe were studied in the selective catalytic oxidation of ammonia (NH3-SCO) and selective catalytic reduction of NO_x_ with ammonia (NH_3_-SCR). Based on recorded results, the conversion of compounds—NH_3_ for NH_3_-SCO and NH_3_/NO_x_ for NH_3_-SCR—were calculated and are presented in Figure 7 and Figure 8, respectively.

The ammonia conversion in the NH_3_-SCO process is rather unaffected by the temperature of the process and, regardless of the material’s composition, does not exceed 20%. This value is too low to treat the Fe-modified Ca-rich and Sr-rich materials as the perspective catalyst, and it can be postulated that in the applied temperature range, these materials are ineffective in the NH_3_-SCO process.

Contrary to the results in the NH_3_-SCO process, all of the studied materials were found to be active in the NH_3_-SCR process, where the reduction of nitrogen oxides with ammonia takes place. Thus, the simultaneous increase in the conversion of both NO_x_ and NH_3_ should process what is clearly seen in the results presented in Figure 8.

The NH_3_ and NO_x_ conversion over all Fe-modified materials starts at approx. 275 °C and gradually increases with the temperature reaching the highest values (97% for NH_3_ conversion) and (94% for NO_x_ conversion) for the Ca_0.8_s_5Fe material at 350 °C. It should be noted that these values are only slightly lower for the Ca_0.8_n_5Fe material (96% and 84%, respectively) but are significantly lower for both Sr-rich materials—Sr_0.8_s_5Fe (62% for NH_3_ conversion and 52% for NO_x_ conversion) and Sr_0.8_n_5Fe (73% for NH_3_ conversion and 63% for NO_x_ conversion). Generally, it should be underlined that the values of conversion of compounds (NH_3_/NO_x_) and the way they change with temperature are similar for stochiometric and non-stoichiometric materials. However, the higher amount of Ca in the system leads to a significant increase in the NH_3_ and NO_x_ conversion since these parameters are more than 20% higher for Ca-rich materials than for Sr-rich materials. The explanation for the higher catalytic activity of Ca-rich materials is most likely the possibility of better ammonia adsorption on their surface, which results from the weaker basic chemical nature of calcium in comparison to strontium and is connected with reaction mechanisms of both reactions.

The NH_3_-SCO and NH_3_-SCR processes aim to convert the ammonia (NH_3_) and nitrogen oxides (NO_x_), respectively, to harmless nitrogen (N_2_) and water vapour (H_2_O). The total conversion and the selectivity toward the desired product can be described by Equation (1) (for NH_3_-SCO) and Equation (2) (for NH_3_-SCR):

(1) $$4{NH}_{3}+3O_{2}\to 2N_{2}+6H_{2}O$$

(2) $$4{NH}_{3}+NO+O_{2}\to 4N_{2}+6H_{2}O$$

The mechanism of NH_3_-SCO is highly influenced by the type of catalyst [55,56]; however, the initial and crucial step in the oxidation process is adsorption of ammonia (Equation (3)), which is followed by the formation of intermediate species: NH_2_* (Equation (4)) [57,58]. For the oxide-based catalysts, the most probable mechanism of NH_3_ oxidation is the internal selective reduction (i-SCR) pathway [55,56]. This reaction pathway involves interactions between the adsorbed intermediates and the NO_x(ad)_ (Equation (6)), which are formed through subsequent oxidation of adsorbed NH_2_* (Equation (5)).

(3) $${NH}_{3(g)}\to {NH}_{3(ad)}$$

(4) $${NH}_{3(ad)}+O^{*}\to {NH}_{2}^{*}+{OH}_{\left(ad\right)}$$

(5) $${NH}_{2}^{*}+O_{2}\to {NO}_{x(ad)}$$

(6) $${NH}_{2}^{*}+{NO}_{x(ad)}\to N_{2}+H_{2}O$$

The NH_3_-SCR mechanism is thought to follow a similar pathway [57,58,59,60]. However, in this case, the NO_x(g)_ directly adsorbs onto active sites instead of being formed via NH_3(ad)_ activation into intermediate forms. Consequently, both, NH_2_^*^ and NO_x(ad)_ can interact, giving the N_2_ and H_2_O (Equation (6)). Simultaneously, adsorbed NO_x(ad)_ might form additional intermediate species, such as NO_3(ad)_ and NO_2(ad)_ (Equation (7)). These intermediates can also interact with activated NH_2_^*^ leading to the formation of N_2_ and H_2_O (Equation (8)).

(7) $${NO}_{x(ad)}+O^{*}\to {NO}_{3(ad)}+{NO}_{2(ad)}$$

(8) $${NO}_{3(ad)}+{NO}_{2(ad)}+{NH}_{2}^{*}\to N_{2}+H_{2}O$$

The similarities between the NH_3_-SCO and NH_3_-SCR mechanisms, along with their complementary pathways, suggest that both processes could occur over the SCR catalysts. However, our results show that the studied materials show poor activity as catalysts of NH_3_-SCO, with the NH_3_ conversion below 20%. This low catalytic efficiency can be explained by the limited formation of NO_x(ad)_ species (Equation (5)), which are crucial for the NH_3_ oxidation. In contrast, the studied materials demonstrate high catalytic efficiency in the NH_3_-SCR process because the NO_x_ can be directly adsorbed onto active sites, eliminating the necessity of NH_2_* oxidation to NO_x(ad)_ (Equation (5)).

The high conversion of compounds is only one of the requirements for catalysts in the NH_3_-SCR process. The high selectivity to N_2_, the most desired product in the reduction of NO_x_ with ammonia, is also a crucial factor. Thus, based on the recorded data, the selectivity to N_2_ and N_2_O, which was also identified as one of the products in the NH_3_-SCR process, was calculated and is presented in Figure 8. For Ca-rich materials, only a small N_2_O amount (3–4%) was observed, and the selectivity to N_2_ was 96–97% in the entire temperature range. For Sr-rich materials, the selectivity to N_2_ is also relatively high (87–89% at 250 °C and 94–96% at 350 °C). Still, it is lower than for Ca-rich materials, and the amount of undesired product (N_2_O) is also higher, especially at lower temperatures. It should be underlined that the deficiency in Ca/Sr sublattice, resulting in formation of vacancies in the A position of the perovskite structure, does not play an essential role in the NH_3__SCR process since selectivity to N_2_/N_2_O and conversion of NH_3_/NO_x_ is comparable for both Ca-rich (Ca_0.8_s_5Fe and Ca_0.8_n_5Fe) and both Sr-rich (Sr_0.8_s_5Fe and Sr_0.8_n_5Fe) materials. On the other hand, selectivity is influenced by the calcium to strontium ratio in the systems since the quantity of undesired products is higher in Sr-rich materials than in Ca-rich ones. Thus, the results of the catalytic test imply that the catalytic performance of the materials is mainly determined by the amount of Ca in the system rather than the oxidation state of iron incorporated into the perovskite structure.

## 3. Materials and Methods

### 3.1. Materials Synthesis

Materials based on the CaTiO_3_-SrTiO_3_ system, with varying iron and calcium concentrations, were synthesised using a modified citrate method. The general formula of samples was as follows: Sr_0.2_Ca_0.8_Ti_1−y_Fe_y_O_3_ and Sr_0.8_Ca_0.2_Ti_1−y_Fe_y_O_3_, where Fe was incorporated at different concentrations —1, 2, or 5 mol.% relative to Ti in the system. Furthermore, samples with 5% iron content were also obtained with a 5% deficiency in the A-site (Ca/Sr) for which the formulas are (Sr_0.2_Ca_0.8_)_0.95_Ti_0.95_Fe_0.95_O_3_ and (Sr_0.8_Ca_0.2_)_0.95_Ti_0.95_Fe_0.95_O_3_. The first series is treated as “stoichiometric” materials (and marked with the s symbol on the labels), while the second as “non-stoichiometric” materials (marked with the n symbol on the labels). The synthesis began by dissolving citric acid, acting as a complexing agent, in methanol within a glass container at 60 °C under continuous stirring. Afterwards, titanium(IV) isopropoxide (Acros Organics) was added, followed by a remaining portion of citric acid. Subsequently, aqueous solutions of strontium nitrate (concentration 1.12 mol dm^−3^ prepared based on Sr(NO_3_)_2_, Aldrich), calcium nitrate (concentration 1.05 mol dm^−3^ prepared based on Ca(NO_3_)_2_·4H_2_O, POCH) and iron(III) nitrate (concentration 1.14 mol dm^−3^ prepared based on Fe(NO_3_)_2_·6H_2_O, Acros) were introduced into the mixture. The concentrations of strontium, calcium and iron nitrate solutions had been previously established using classical chemical analysis methods. The molar ratio between the sum of Ti, Sr, Ca and Fe ions to citric acid was maintained at 2:3. Lastly, polyvinyl alcohol (4%) was added to the mixture to enable potential esterification reactions. The resulting sol was heated to 120 °C, followed by drying at 220 °C for 10 h. Once cooled, the materials were ground in an agate mortar and calcined in an air atmosphere at 900 °C for 3 h. Finally, the synthesised materials were analysed further.

### 3.2. Methods

X-ray diffraction (XRD) measurements were conducted over a 2Θ angle range of 10° to 90° using a Phillips X’Pert Pro diffractometer (PANanlitycal, Malvern, UK) with CuKα radiation (λ = 1.5406 Å) and a step size of 0.008°. The phase composition of materials was analysed through Rietveld refinement using HighScore Plus software (version 3.0.4). Crystallographic data for the analysis were obtained from the ICCD database, referencing phases such as SrTiO_3_ (Pm-3 m, No. 98–005–6717), Sr_0.65_Ca_0.35_TiO_3_ (I4/mcm, ICCD No. 98-009-4572), CaTiO_3_ (Pnma, ICCD No. 98-016-2910), TiO_2_ (rutile, P42/Mnm, ICCD No. 98-008-5492) and TiO_2_ (anatase, I41/amd, ICDD No. 98-015-4607). The GoF (Goodness of Fit) parameter ranged from 0.82 to 1.52 for all samples, indicating a good fit between the observed and calculated patterns. The diffraction data analysis provided information on the qualitative and quantitative phase composition of the materials after calcination and the materials after reduction in hydrogen-containing gas.

For microscopic analysis of the powdered samples, a Tescan Vega scanning electron microscope (SEM) (Tescan Group Ltd., Brno, Czech Republic), equipped with a tungsten cathode was utilised. Micrographs were captured in a combined secondary electron (SE) and backscattered electron (BSE) mode, using an acceleration voltage of 15 KeV. Energy-dispersive X-ray spectroscopy (EDS: EDAX) was employed for chemical composition analysis. Prior to imaging, the samples were gold-sputtered to enhance electron conductivity. Images were taken from random areas of the samples to ensure an unbiased analysis, avoiding the focus on anomalies or artefacts.

Temperature-programmed reduction (TPR) and temperature-programmed oxidation (TPOx) measurements were performed using a ChemiSorb 2750 apparatus (Micromeritics Instrument Corporation, Norcross, GA, USA). The samples, after calcination, weighing approximately 0.2 g, were placed in a quartz reactor and subjected to reduction and oxidation cycles according to the following scheme: I reduction → I oxidation → II reduction. The reduction was conducted in a 5% H_2_/Ar atmosphere, while oxidation was performed in a 5% O_2_/Ar atmosphere, both at a flow rate of 40 mL min^−1^. The temperature was increased from room temperature to 900 °C at a rate of 10 °C min^−1^. Before TPR measurements, samples were pre-treated in helium and then stabilised in a 5% H_2_/Ar mixture to ensure a stable TCD detector baseline. Between the TPR and TPOx cycles, the system was flushed with helium at room temperature to ensure proper gas exchange.

X-ray absorption spectra (XAS) were collected at the ASTRA beamline of the National Synchrotron Radiation Centre (SOLARIS) in Krakow. Measurements were performed on powdered materials using transmission mode for elements exceeding 20 mol.% (for Ti) and fluorescence mode when the molar fraction was below 20 mol.% (in the case of Fe). Spectra were recorded for the K edge of Ti and Fe. The resulting XANES spectra were processed and normalised using Athena software included in the Demeter 0.9.26 package.

The catalytic activity of the prepared materials for selective catalytic oxidation of ammonia (NH_3_-SCO) and selective catalytic reduction of NO_x_ with ammonia (NH_3_-SCR) was evaluated using a fixed-bed quartz microreactor system. The reactions were carried out at atmospheric pressure across a 200–350 °C temperature range, with isothermal steps every 25 °C. A quantity of 200 mg of catalyst with particle sizes between 160–315 μm was used for each experiment. Before measurements, the samples were activated in an airflow of 100 mL/min at 200 °C for 60 min. In the NH_3_-SCO process, a gas mixture containing 0.035 mol.% NH_3_ diluted in N_2_ and synthetic air (total flow of 100 mL/min) was introduced into the reactor. In the NH_3_-SCR process, the gas mixture included 0.035 mol.% NH_3_ in N_2_, 0.035 mol.% NO in He, and 2 mol.% O_2_ in N_2_ (total flow of 100 mL/min). The detection of all gaseous reactants and products, including nitric oxide (NO), nitrogen dioxide (NO_2_), nitrous oxide (N_2_O), and water vapour, was conducted using a Fourier-transform infrared (FT-IR) spectrometer (Antaris IGS, Nicolet, Prague, Czech Republic). Based on the recorded spectra, the ammonia conversion in the NH_3_-SCO and NH_3_-SCR processes and the conversion of NO_x_ in the NH_3_-SCR process were calculated together with the selectivity towards specific products—N_2_ and N_2_O. Details of the calculation procedure are written in [4].

## 4. Conclusions

The effect of iron doping and the effect of non-stoichiometry introduced into the Ca/Sr sublattice on the structural properties and susceptibility of the redox process of the CaTiO_3_-SrTiO_3_-mixed perovskite (Ca-rich and Sr-rich materials) were analysed, together with the evaluation of the possibility of applying of the materials as a catalyst in NH_3_-SCR and NH_3_-SCO processes. The following assumptions can be made as a result of the provided studies:

1. Iron is fully incorporated into both Ca-rich and Sr-rich materials (up to 5 mol.%) without forming any Fe-rich phases.
2. The average oxidation state of iron incorporated into the perovskite structure is higher than +3, suggesting iron’s presence on various oxidation states, including +4.
3. The reduction process of Fe-modified Ca-rich and Sr-rich materials is partially reversible, especially for the small amount (1 and 2 mol.%) of dopant.
4. The iron-doped mixed CaTiO_3_-SrTiO_3_ system is an effective catalyst in NH_3_-SCR process, while it is inactive in NH_3_-SCO.
5. The Ca-rich materials are more promising catalysts than Sr-rich materials in NH_3_-SCR since the conversion of NH_3_/NO_x_ and selectivity to N_2_ for Ca-rich materials is around 20% higher than for Sr-rich materials.
6. Calcium’s weaker basic chemical nature than strontium results in higher catalytic activity of Ca-rich materials in the NH_3_-SCR process.

## Figures and Tables

**Table 1:** Nominal composition of the synthesised materials and corresponding sample labels.

**Ca-Rich Materials**				
sample	Sr_0.2_Ca_0.8_Ti_0.99_Fe_0.01_O_3_	Sr_0.2_Ca_0.8_Ti_0.98_Fe_0.02_O_3_	Sr_0.2_Ca_0.8_Ti_0.95_Fe_0.05_O_3_	(Sr_0.2_Ca_0.8_)_0.95_Ti_0.95_Fe_0.05_O_3_
label	Ca_0.8_s_1Fe	Ca_0.8_s_2Fe	Ca_0.8_s_5Fe	Ca_0.8_n_5Fe

**Sr-Rich Materials**				
sample	Sr_0.8_Ca_0.2_Ti_0.99_Fe_0.01_O_3_	Sr_0.8_Ca_0.2_Ti_0.98_Fe_0.02_O_3_	Sr_0.8_Ca_0.2_Ti_0.95_Fe_0.05_O_3_	(Sr_0.8_Ca_0.2_)_0.95_Ti_0.95_Fe_0.05_O_3_
label	Sr_0.8_s_1Fe	Sr_0.8_s_2Fe	Sr_0.8_s_5Fe	Sr_0.8_n_5Fe

**Table 2:** The phase composition of Fe-modified Ca-rich and Sr-rich materials.

Sample	Phase Composition/Mass.%			
	SrTiO_3_	t-Sr(Ca)TiO_3_	o-CaTiO_3_	TiO_2_
Ca-rich materilas				
Ca_0.8_s_1Fe	0	0	98.6	1.4
Ca_0.8_s_2Fe	0	0	98.9	1.1
Ca_0.8_s_5Fe	0	0	100	0
Ca_0.8_n_5Fe	0	0	100	0
Sr-rich materilas				
Sr_0.8_s_1Fe	24.1	72.0	0	3.9
Sr_0.8_s_2Fe	26.1	69.9	0	4.0
Sr_0.8_s_5Fe	35.7	64.3	0	0
Sr_0.8_n_5Fe	37.9	62.1	0	0

## Data Availability

The datasets analysed during the reported study will be made available upon reasonable request.
